# Offloading Interventions for the Management of Charcot Neuroarthropathy in Diabetes

**DOI:** 10.1177/24730114251315670

**Published:** 2025-02-10

**Authors:** Thomas Berhane, Kanakamani Jeyaraman, Mark Hamilton, Henrik Falhammar

**Affiliations:** 1 Department of Medicine, Royal Darwin Hospital, Darwin, NT, Australia; 2 Logan Endocrine and Diabetes Services, Logan Hospital, Logan, QLD, Australia; 3 Department of Vascular Surgery, Royal Darwin Hospital, Darwin, NT, Australia; 4 Menzies School of Health Research, Darwin, NT, Australia; 5 Department of Endocrinology, Karolinska University Hospital, Stockholm, Sweden; 6 Department of Molecular Medicine and Surgery, Karolinska Institutet, Stockholm, Sweden

**Keywords:** Charcot foot, diabetes osteoarthropathy, patella tendon bearing cast, removable offloading

## Abstract

**Background:**

The use of a nonremovable patellar tendon bearing (PTB) cast in Charcot neuroarthropathy (CA) has not been well studied. We describe the offloading devices, including PTB cast used in our setting for the treatment of CA.

**Methods:**

We performed a retrospective observational study on patients with CA and diabetic foot ulcer (DFU) presenting to the multidisciplinary foot clinic at Royal Darwin Hospital, between January 2003 and June 2015. Various immobilization and offloading methods used in CA treatment and their outcomes were analyzed.

**Results:**

Ninety-three cases of CA were included. PTB cast (n = 76) and a variety of custom-made removable devices (n = 17) were used for initial offloading. Patients treated with PTB casts were allowed to fully weightbear on the affected limb, as tolerated. Initial offloading was continued until the joint stabilized and ulcer healed (6.5±1.9 months), and then patients were transitioned to various orthotic devices and then to accommodative footwear. At the end of the whole offloading treatment (median duration 13.1 months; range 10-24), patients treated with PTB initially had better outcomes compared with patients treated with removable devices.

**Conclusion:**

Immobilization using PTB casting was an effective offloading method for CA with DFU. With our offloading regimen, Indigenous and non-Indigenous patients had similar outcomes.

**Level of Evidence:**

Level III, retrospective cohort study.

## Graphical abstract

**Visual Abstract:**
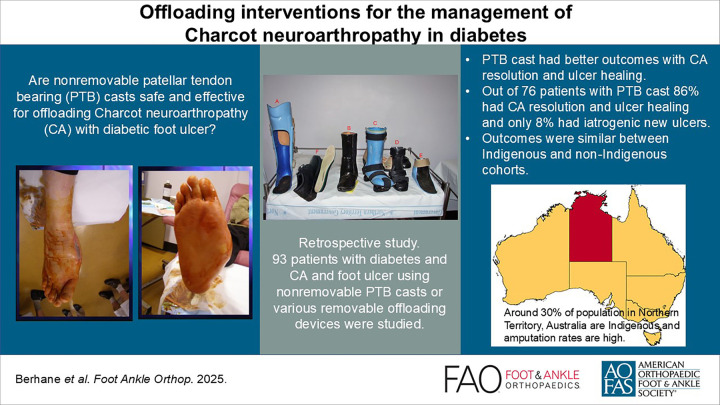
This is a visual representation of the abstract.

## Introduction

Charcot neuroarthropathy (CA) is a rare complication of diabetes mellitus, affecting 0.2% to 3% of all patients with diabetes mellitus.^2,10,12,26,30^ People with CA often have multiple system disease,^24^ and treating this complex disease in a highly comorbid population is a challenge. Immobilization and offloading are key to limiting structural damage to the foot and ankle.^2,14,21,36^ Total-contact cast (TCC) is generally accepted as the mainstay treatment for CA.^2,13,14,22,26,35^ However, there is no consensus on the type of immobilization (removable vs nonremovable) and offloading (weightbearing vs nonweightbearing).^4,23,29^

In our multidisciplinary foot clinic (MDFC), we have successfully used a nonremovable patellar tendon weightbearing (PTB) cast to stabilize joints affected by CA with associated diabetic foot ulcer (DFU). The concept of PTB cast is not widely accepted, and the exact mechanism of how it offloads the foot remains to be studied. There are limited data to support the use of PTB casts in patients with CA. In our population with a significant proportion of Indigenous patients with limited access to health care and nonadherence, we found that a PTB cast was effective to improve adherence. This study aims to (1) describe the different offloading devices used in our setting for the treatment of CA and (2) evaluate their efficacy in achieving and maintaining joint stability and healing of DFU.

## Materials and Methods

The study was conducted at the Royal Darwin Hospital (RDH), the only tertiary hospital in the Northern Territory (NT) of Australia, with a catchment area covering 1.35 million km^2^ but with a population of only 246 000. Many NT inhabitants live remotely, and around 30% are Indigenous.^17^

All patients ≥18 years old with CA and DFU, presenting for the first time to the MDFC at RDH between January 2003 and June 2015, were included. The study methodology was described in detail previously.^3,18^ Patients were treated and regularly reviewed by the MDFC team, until joint stabilization and ulcer healing was achieved. CA was confirmed with radiology. If characterized by swelling, warmth, and erythema, a clinical diagnosis of acute CA was made. Patients presenting with foot deformity were diagnosed as chronic CA. Access to magnetic resonance imaging (MRI) was limited during the period of the study.

Offloading modalities were discussed and tailored to individual patients based on clinical findings, imaging, ulcer characteristics, patient mobility, and preference. The offloading devices used in the MDFC were PTB cast, PTB orthosis, High Walking Boot (HWB), Darwin Walker (DW), Ankle Foot Orthosis (AFO), Integrated Shoe with AFO (ISAFO), and Orthopaedic Shoe (OS). All our offloading devices, both removable and nonremovable, were designed and applied by a single orthotist (author T.B.). Our preferred initial offloading treatment was PTB cast. Removable offloading devices were used for initial offloading only if (1) the deformity could not be accommodated by orthotic and shoe combinations, (2) there was excessive swelling, or (3) it was patient preference.

The PTB cast was fiberglass designed to reduce plantar pressure by facilitating load distribution on the plantar surface of the foot. To reduce the risk of cast-related complications, we used multiple layers of Soffban padding and a Soffban waterproof cast liner around the head of fibula, hamstring tendon, and other bony prominences. A PTB cast suspends weight around the patellar tendon reaching the plantar surface of the foot, achieved by an intimate fit of the cast around the patella through meticulous molding. We used this method of PTB casting for all patients with CA and DFU, both acute and chronic. Patients were allowed to fully weightbear on the limb as tolerated, aided by a casting shoe and other assistive devices, if needed. All PTB casts were applied using the material and technique described in our previous study.^3^

Independent mobility and postural stability in the cast were assessed and appropriate gait training was provided. Casts were checked regularly in the MDFC and replaced as necessary, often at 1-2-week intervals. In patients with infected wounds, casts were removed weekly for inspection and debridement. In some noninfected wounds, PTB casts were retained up to 10 weeks or more, particularly in patients from remote areas. Imaging of the foot and ankle were conducted frequently in the early phase and then as dictated by the clinical picture.

**Figure 1.**
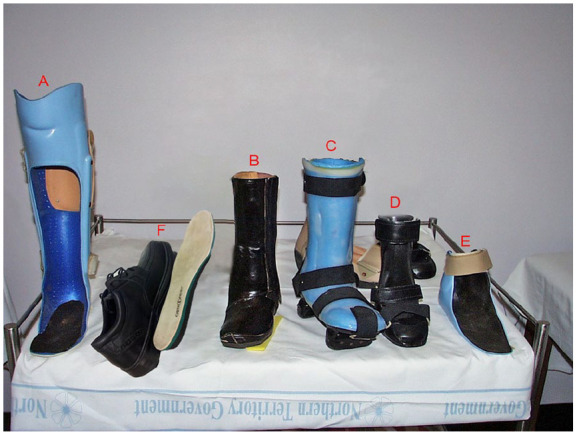
Custom-made offloading devices (A, B, C, D, and E) and commercial foot wear (F). (A) Patella tendon bearing orthosis (PTB orthosis). (B) High walking boot (HWB). (C) Darwin walker (DW). (D) Integrated shoe and ankle foot orthosis (ISAFO). (E) Ankle foot orthosis (AFO). (F) Orthopaedic shoe (OS).

Following PTB casting, patients were introduced to one of the alternative removable offloading devices (Figure 1A-E) and later to footwear (Figure 1F). The timing of the transition was determined both by clinical and radiologic examination. We considered skin temperature equilibrium between limbs as a reliable sign of coalescence and sufficient structural stability to permit transition. An infrared thermometer to measure foot temperature was not used. During this transition period, patients were followed up at 2- to 3-month intervals, until CA resolution was complete and wound closure was achieved. The time of resolution of CA was defined as the time when the patient was ambulant in normal or orthotic footwear.

The study was approved by the Human Research Ethics Committee of the NT Department of Health and Menzies School of Health Research (HREC-2015-2324). Statistical analyses were performed using Stata, version 14.2. Missing data were random and were handled by case deletion. Data were described as mean ± SD or median (range) as appropriate. Unpaired *t* test, Wilcoxon-Mann-Whitney *U* test, and χ^2^ test were used to assess the differences between groups.

## Results

**Figure 2.**
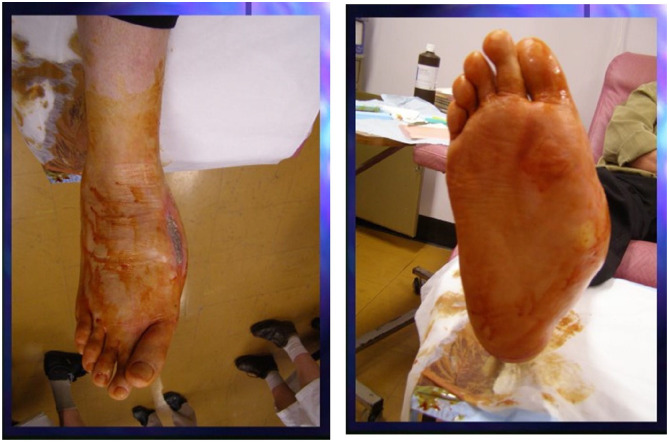
Typical acute Charcot neuroarthropathy with foot ulcer.

Of the 513 patients treated for DFU in the MDFC during the study period, 18.1% (93/513) had CA. Eleven patients had acute CA with foot deformities (Figure 2). Bilateral CA was present in 4 patients. The most common site was the midfoot, at 39% (36/93).

PTB cast was the initial offloading device in 76 patients, and removable offloading was used in 17 cases. HWB and ISAFO were used as initial offloading options for patients (n = 11) who were considered unsuitable for other types of offloading because of severe foot ankle deformity. A DW was used for patients (n = 4) with significant deformity like rocker-bottom deformity and in patients who declined PTB cast. PTB orthosis was used in 2 patients for initial offloading. AFO was never used in the initial offloading.

Table 1 compares the baseline characteristics and clinical outcomes of the patients treated with PTB cast and removable devices. PTB cast was the most common offloading device used in Indigenous patients, particularly for those who reside in remote areas. The mean length of initial offloading and CA resolution was lower in patients treated with PTB than with removable devices (6.4±1.6 vs 7.1±2.7 months). With our initial PTB offloading treatment, 65 of 76 patients had CA resolution and ulcer healing. Three patients progressed to amputation. With the small numbers available, no significant differences could be detected in the outcomes after the initial offloading between the PTB and the removable devices group.

**Table 1.** Comparisons Between Patients With Charcot Neuroarthropathy and Diabetic Foot Ulcer Using Patella Tendon Bearing (PTB) or Removable Orthosis.

Patient Characteristics	PTB (n = 76)	Removable (n = 17)	*P* Value
Age at initial presentation, y, mean ± SD	51.0±11.1	63.1±9.1	<.001
Females	32 (42.1%)	5 (29.4%)	.334
Indigenous	43 (56.6%)	5 (29.4%)	.04
Remote	27 (35.5%)	0 (0%)	.004
BMI, mean ± SD	27.2±6.2	28.1±6.5	.60
Current smokers	40 (52.6%)	10 (58.8%)	.643
Type 2 diabetes	70 (92.1%)	17 (100%)	.201
Duration of diabetes, y, median (IQR)	14 (5-20)	12 (7-17)	.14
Glycated hemoglobin, %, mean ± SD	9.0±2.3	9.5±3.4	.73
Insulin treatment	49 (64.5%)	9 (52.9%)	.375
Loss of protective sensation	72 (94.7%)	13 (76.5%)	.015
Retinopathy	46 (63.9%)	9 (52.9%)	.776
Nephropathy	51 (61.1%)	8 (47.1%)	.121
Dialysis	15 (19.7%)	3 (17.6%)	.844
Peripheral arterial disease	32 (42.1%)	8 (47.1%)	.709
Hypertension	72 (94.7%)	17 (100%)	.334
Previous ulcer	67 (88.2%)	15 (88.2%)	.993
Previous amputation	38 (50.0%)	4 (23.5%)	.047
Site of Charcot			.015
Forefoot	25 (32.9%)	3 (17.6%)	
Midfoot	25 (32.9%)	11 (64.7%)	
Hindfoot and ankle	26 (34.2%)	3 (17.6%)	
Late Charcot	6 (7.9%)	5 (29.4%)	.013
Wagners grade			.074
Grade 1	16 (22.2%)	8 (47.1%)	
Grade 2	18 (25.0%)	2 (11.7%)	
Grade 3	5 (6.8%)	6 (35.3%)	
Osteomyelitis confirmed with imaging	45 (59.2%)	8 (47.1%)	.360
Duration of offloading, mo, mean ± SD	6.4±1.6	7.1±2.7	.058
Initial outcome (at the end of initial offloading)			.684
Joint stabilized and ulcer healed	65 (87.8%)	15 (88.2%)	
Joint stabilized and ulcer not healed Amputation	6 (8.1%)3 (4.1%)	1 (5.9%) 0 (0%)	
Lost to follow-up	0 (0%)	1 (5.9%)	
Final outcome (at the end of offloading treatment)			.002
Stable	52 (68.4%)	7 (41.2%)	
Amputation/wheelchair bound	17 (22.6%)	2 (11.7%)	
Death	5 (6.6%)	6 (35.3%)	
Lost to follow-up	2 (2.6%)	2 (11.7%)	

Abbreviations: BMI, body mass index; IQR, interquartile range.

The initial offloading was followed by transition to various orthotic devices over 3.5±1.9 months and then a gradual transfer to an accommodative footwear over 6.1±1.8 months. The median duration of the whole offloading treatment was 13.1 (range, 10-24) months. At the end of the whole offloading treatment, patients treated with PTB initially, had better outcomes with stable joints (52/76 vs 7/17, *P* = .002) and lower mortality (5/76 vs 6/17, *P* = .002).

The most common complications resulting from the use of PTB casting were dermal abrasions and iatrogenic ulceration, with 6 new ulcers in 76 patients and 760 casts. These were minor, reversible, and did not interfere with the treatment plan. Another problem observed was maceration due to wound exudate and the hot, humid tropical climate. This significantly improved with the use of Soffban waterproof liner. We are currently trying alternative casting materials like Scotchcast with more climate-friendly characteristics.

A large proportion (48/93) of our patients were Indigenous. Indigenous patients were younger and predominantly female, and about a half (23/48) were from remote areas (Table 2). About 1 in 3 (18/48) were on dialysis. Indigenous patients had lower body mass index (BMI). The midfoot was the predominant site for CA in the non-Indigenous, whereas in Indigenous patients midfoot and hindfoot were equally affected. This could be explained by the higher BMI in the non-Indigenous patients. The duration of initial offloading treatment was slightly longer in the Indigenous group (6.8±2.1 vs 6.2±1.6 months). Regardless of these differences, the final outcomes of CA resolution and DFU healing were similar in Indigenous (33/48) and non-Indigenous (26/45) patients.

**Table 2.** Clinical Characteristics, Management Outcomes of 93 Patients With Acute and Chronic Charcot Neuroarthropathy, and Ankle and Diabetic Foot Ulcer.

Patient Characteristics	Non-Indigenous (n = 45)	Indigenous (n = 48)	*P* Value
Age at initial presentation, y, mean ± SD	51.0 ± 11.1	63.1 ± 9.1	<.001
Female	9 (20.1%)	28 (58.3%)	<.001
Remote	4 (8.9%)	23 (47.9%)	<.001
BMI	28.8 ± 6.2	26.1 ± 6.4	.02
Current smokers	19 (42.2%)	10 (58.3%)	.643
Type 2 diabetes	40 (88.9%)	47 (97.9%)	.07
Duration of diabetes, y, median (IQR)	14 (5-20)	12 (7-17)	.13
Glycated hemoglobin, %	9.1 ± 2.3	9.2 ± 2.6	.51
Insulin treatment	31 (68.9%)	27 (56.2%)	.209
Neuropathy	39 (86.7%)	46 (95.8%)	.115
Retinopathy	19 (42.2%)	36 (75.0%)	.018
Nephropathy	19 (42.2%)	40 (83.3%)	<.001
Dialysis	3 (6.7%)	15 (31.2%)	.003
Peripheral arterial disease	19 (42.2%)	21 (43.7%)	.882
Hypertension	41 (91.1%)	48 (100%)	.03
Previous ulcer	40 (88.9%)	42 (87.5%)	.836
Previous amputation	19 (42.2%)	23 (47.8%)	.581
Site of Charcot			.015
Forefoot	13 (28.8%)	15 (31.3%)	
Midfoot	25 (55.6%)	11 (22.9%)	
Hindfoot and ankle	7 (15.6%)	22 (45.8%)	
Late Charcot	6 (13.3%)	5 (10.4%)	.663
Wagners Grade			.074
Grade 1	16 (41.0%)	8 (50.0%)	
Grade 2	18 (46.2%)	2 (12.5%)	
Grade 3	5 (12.8%)	6 (37.5%)	
Osteomyelitis confirmed with imaging	20 (44.4%)	20 (41.7%)	.787
Duration of initial offloading, y, mean ± SD	6.2 ± 1.6	6.8 ± 2.1	.06
Initial outcome (at the end of initial offloading)			.113
Joint stabilized and ulcer healed	37 (88.1%)	43 (89.5%)	
Joint stabilized and ulcer not healed	2 (4.8%)	5 (10.5%)	
Amputation	3 (7.1%)	0 (0.0%)	
Lost to follow-up	3 (6.7%)	0 (0.0%)	
Final outcome (at the end of initial offloading)			.706
Joint stabilized and ulcer healed	26 (57.8%)	33 (68.8%)	
Joint stabilized and ulcer not healed	10 (22.2%)	9 (18.8%)	
Amputation	6 (13.3%)	5 (10.4%)	
Lost to follow-up	3 (6.7%)	1 (2.1%)	

Abbreviations: BMI, body mass index; IQR, interquartile range.

## Discussion

TCC is considered the gold standard for management of acute CA.^26^ Other nonremovable offloading can also be effective and safe to prevent ulcers in acute CA.^9,25^ There is no consensus on the use of weightbearing or nonweightbearing casts, the choice of the offloading device, and the duration of offloading in the treatment of CA.^23^

To our knowledge, there are no published studies describing the use of a nonremovable PTB cast as an offloading device in the treatment of CA in patients with DFU. Katz et al have shown that in plantar neuropathic DFUs, an irremovable PTB cast is equally effective to TCC with the added advantage of ease of use, faster application, and less expensive.^20^ In our patients with complex foot deformities with no other offloading or surgical options, joint stability and ulcer healing were better with the nonremovable PTB cast. The Australian Indigenous population is known to have poor outcomes from DFU. With our offloading regimen, CA resolution and DFU healing were similar between the Indigenous and the non-Indigenous groups.

A PTB cast suspends weight from around the horizontal axis of patellar tendon, whereby, in TCC loading occurs in the proximal tibia shank cast wall. We believe that a significant reduction in the load under midfoot occurs with a PTB cast. A PTB cast covers more interface area than TCC and takes more load from the horizontal curve of the patella tendon, resulting in an increased patella area load and decreased plantar foot surface load. The PTB cast is similar to fitting a prosthetic limb to a transtibia amputee with bony prominence at the end of the stump, where the load is directed to only the horizontal patellar tendon axis without contact with the distal end of the stump. Using dynamic plantar pressure analysis, a study on 5 healthy subjects reported the unloading effect of PTB cast by producing a space between the sole of the foot and the cast by 1-, 2-, and 3-cm cushioning was 60%, 80%, and 98%, respectively.^32^ Further mechanistic studies are needed to understand the exact mechanism of how the PTB cast unloads the foot.

In various studies, the duration of TCC for the treatment of CA ranged from 6 to 12 months.^7,22,23,29^ In our patients, the duration of PTB casting before returning to footwear was longer than the TCC cast reported by some studies.^2,29^ The longer offloading duration and healing time in our study were likely related to delay in presentation, diagnosis, and offloading treatment. Often, acute CA was misdiagnosed as gout, arthritis, or infection. At initial presentation to our clinic, most patients had undiagnosed CA for 1-6 months, resulting in progression to foot deformity, ulceration, and loss of function. Studies show that when CA is diagnosed and treated within 1 month of symptom onset, approximately 70% heal without foot deformity with an average immobilization of 4 months whereas patients diagnosed after 2 months of symptom onset needed an average immobilization time of 5 months, with only 30% healing without foot deformity.^6^ Other factors such as preexisting foot deformity, prior amputation, osteomyelitis, and other diabetes-related complications may have influenced the treatment duration in our study population. Insufficient duration of offloading is known to result in deformity or deterioration of an existing foot deformity.^7^ Our observation was similar, and all patients who had less than 6 months of initial PTB cast had a relapse of CA and needed further recasting treatment for an additional 3-6 months.

In our setting, patients in a PTB cast were allowed unrestricted full weightbearing throughout the duration of PTB cast. A strict nonweightbearing approach was impractical for most of our patients because of a variety of socioeconomic, environmental, and geographical factors. In our experience, full weightbearing with the PTB cast is effective and practical in meeting patients’ daily needs, leading to higher patient satisfaction and good compliance rates. The impact of mobility limitation leads to a cascading effect on a variety of quality-of-life domains for both patients and their caregivers.^15^ Despite this, strict nonweightbearing for 3 months is recommended by some clinicians until the acute inflammatory event subsides,^8^ whereas others allow weightbearing as tolerated from commencement of treatment without any deleterious effects.^9,12,25^ In our observation, full weightbearing in a PTB cast did not result in increased deformity or ulceration and did not prolong time of immobilization. No harm occurred with free ambulation during the time of casting, except for some minor and reversible complications such as skin irritation.

In the literature, full weightbearing in a cast is recommended for forefoot or midfoot CA, with nonweightbearing recommended for rearfoot CA.^34^ Our offloading principles were the same for all CA sites and we did not find any difference between the CA site and offloading types. After the treatment period, 67.9% of forefoot CA, 62.5% of midfoot CA, 75.0% of hindfoot CA, and 61.5% of ankle CA achieved joint stability. There are no studies reporting if the site of CA affects the time to healing. In our study, the duration of offloading and healing time was similar in the hindfoot and ankle joint (7.0 months) compared with forefoot (6.5 months) and midfoot (6.2 months).

We used several removable offloading devices for initial offloading when deformity could not be accommodated in a PTB cast and as second or third transitional options, after the completion of PTB casting. Among the removable devices, we chose PTB orthosis to stabilize the foot and ankle joints, by transferring the weight to the patellar tendon. A properly fitted PTB orthosis reduces mean peak forces to the distal extremity by 32% to 90%.^16,27^ In our experience, other removable offloading devices such as OS, HWB, DW, and AFO did not reduce plantar loads as effectively as a PTB cast or PTB orthosis but were certainly useful in preventing ulcer recurrence and in maintaining joint structural stability. It is well known that the removable offloading devices are not as effective as nonremovable devices,^1^ primarily because of compliance with the removable devices,^5^ and our clinical experience was not any different.

In the current study, AFO was used only as a second transition device. A few patients in the late stage of CA developed ulcers after fitting an AFO, primarily in the prominent bony areas of the foot and ankle. In our patient population, AFO was associated with greater levels of ulcer recurrence and increased the risk of amputation. Further studies are needed to confirm our clinical observation. DW was effective as a bridge in shifting midfoot concentrated peak pressure to the forefoot and hindfoot.

HWB and ISAFO were mainly used in patients with advanced ankle and foot joint deformity for maintaining ankle joint stability and for preventing ulcer recurrence following casting. Both devices were effective for plantar pressure reduction and ulcer healing compared with other footwears. Again, because of small sample size, we cannot comment on the effectiveness of these offloading devices. A study designed with an adequate sample size and formal plantar pressure measurement will be helpful to confirm our clinical observation.

A large proportion of our study population were Indigenous patients with several comorbidities. About 1 in 5 were on dialysis and almost half had a history of LEA. Studies have shown that comorbidities such as renal dysfunction and poor cardiovascular status can affect wound healing.^11,14^ However, with our offloading approach, the outcomes were good, with an amputation rate of 19.1%. Other studies report amputation rates from 1% to 15%.^27,31^ However, all our patients had DFU, and the presence of an ulcer increases the risk of amputation by 12 to 13 times.^31^ Saltzman et al reported a major amputation rate of 28% in patients with ulcers compared with 7% in patients without ulcers.^27^ Several other studies have also reported that when CA is associated with ulceration and infection it can increase the risk for LEA and mortality.^26,28,33^ In our previous study, we have reported that 33.1% of the Indigenous patients vs 31.4% of the non-Indigenous patients had an LEA during 5.8 years of follow-up.^19^ In the current study, the amputation rate at the end of the offloading treatment was 14.3% vs 11% for non-Indigenous and Indigenous patients, respectively.

The strength of our study is the large number of patients with CA and the long duration of follow-up. Patients were treated in a single-center multidisciplinary clinic that manages a high volume of diabetic foot complications. All patients were treated by the same team for the whole duration of the CA treatment. Even though, at initial presentation PTB casts were applied immediately based on clinical diagnosis, for this study we have used diagnostic imaging as standard criteria for the initial diagnosis and later for remission of CA. The limitations relate to its retrospective design. There were missing data regarding previous treatment and clinical history for some patients referred with chronic CA. We have not used infrared dermal thermometry to assess the progress and healing of CA. All our offloading devices were custom designed, based on each patient’s needs and clinical findings. Such a personalized approach to offloading may not be a viable option in other settings, and hence our findings may not be applicable to a wider population.

## Conclusion

In this study, we found that immobilization using nonremovable PTB cast with FWB was an effective offloading method for CA with DFU. Our step-down approach with offloading improved outcomes in our patients with a large proportion of Indigenous patients that are well known to have poor outcomes, including diabetes-related amputations. Treatment outcomes including amputation rates were similar in the Indigenous and non-Indigenous group.

## Supplemental Material

sj-pdf-1-fao-10.1177_24730114251315670 – Supplemental material for Offloading Interventions for the Management of Charcot Neuroarthropathy in Diabetes Supplemental material, sj-pdf-1-fao-10.1177_24730114251315670 for Offloading Interventions for the Management of Charcot Neuroarthropathy in Diabetes by Thomas Berhane, Kanakamani Jeyaraman, Mark Hamilton and Henrik Falhammar in Foot & Ankle Orthopaedics
